# The Role of Perceived Control in the Psychophysiological Responses to Disgust of Subclinical OCD Women

**DOI:** 10.3390/s19194180

**Published:** 2019-09-26

**Authors:** Miguel Ángel Serrano, Vicent Rosell-Clari, Gemma García-Soriano

**Affiliations:** 1 Departamento de Psicobiología, Facultad de Psicología, Universitat de València, 46010 Valencia, Spain; M.Angel.Serrano@uv.es; 2 Departamento de Psicología Básica, Facultad de Psicología, Universitat de València, 46010 Valencia, Spain; Vicente.Rosell@uv.es; 3 Departamento de Personalidad, Evaluación y Tratamientos Psicológicos, Facultad de Psicología, Universitat de València, 46010 Valencia, Spain

**Keywords:** obsessive‒compulsive disorder, heart rate variability, emotion, women, disgust, contamination

## Abstract

Obsessive‒compulsive disorder (OCD), and especially contamination obsessions and washing compulsions, has been related to disgust. However, when its cardiovascular correlates have been studied, contradictory results have been found, including heart rate accelerations and decelerations. The aim of this study is to analyze emotional, cognitive, and cardiovascular responses in nonclinical (control) and subclinical participants with obsessive‒compulsive contamination/washing symptoms when confronted with a disgusting stimulus. Twenty-seven participants (14 subclinical OCD) completed a behavioral avoidance task with a contamination-based stimulus while their heart rate and subjective variables were measured. Results showed heart rate reductions in both samples, whereas subjective measures reflected higher disgust, anxiety, dirtiness, and emotional valence in the subclinical sample. However, at the same time, the sense of dominance was lower in the control group. In conclusion, our results support a heart rate deceleration during exposure to a disgusting stimulus dissociated from the subjective experience.

## 1. Introduction

Obsessive‒compulsive disorder (OCD) is a disorder characterized by recurrent heterogeneous obsessions and repetitive behaviors. Although traditionally associated with fear [1], in recent decades, OCD, and especially contamination obsessions and washing compulsions, has also been related to disgust [2,3,4]. Most of the studies analyzing the role of disgust in OCD have appraised it from a subjective perspective, thus ignoring its physiological correlates [5]. As far as we know, only a few studies [6,7,8,9,10] have analyzed heart rate (HR) correlates in clinical or subclinical OCD samples exposed to disgusting or contaminated stimuli, with divergent results, such as heart rate acceleration, deceleration, or no significant changes. Recently, it has been suggested that the contradictory results could be due to the fact that “few of these studies attempted to differentiate between emotions such as anxiety and disgust” [7].

Since Darwin’s conceptualization, disgust has been considered a basic, universal emotion that has the adaptive function of protecting the body from contact with and the incorporation of harmful elements [11]. Disgust produces nausea and feelings of revulsion [11]. However, the study of this emotion has been ignored in psychophysiological research [12], even though, in general, studies find that there is a different autonomic nervous system (ANS) activation for each of the basic emotions [13].

In this regard, disgust has been related to cardiovascular responses [14]. Specifically, some authors have suggested that cardiovascular disgust reactions are mediated by sympathetic and parasympathetic co-activation [15,16]. Disgust has been associated with low cardiac flexibility, measured by heart rate variability [17] (HRV), an index of brainstem-mediated parasympathetic influences on the heart, associated with adaptive and flexible regulation [18,19]. However, studies are not clear about whether disgust produce increases or decreases in cardiovascular activity. The majority of studies have observed a heart rate deceleration during disgust provocation [20,21,22,23]. Nevertheless, other studies have reported heart rate accelerations associated with exposure to disgusting stimuli [24,25,26]. Moreover, it has been suggested that the physical dimension of disgust elicits enhanced activity of the parasympathetic nervous system (increased HRV), whereas heart rate does not show any changes [27]. These inconsistencies raise the question of why disgust provokes increases, decreases, or does not change cardiovascular activity depending on the study.

There might be different reasons for these inconsistencies. First, differences could be due to the type of disgust provocation method (e.g., images, videos, bodily waste, or blood), which could elicit different physiological and self-reported responses [16]. Along these lines, it has been suggested that images of mutilation elicit heart rate deceleration, suggesting decreased cardiac sympathetic control, whereas images of contamination (e.g., bodily waste) elicit heart rate acceleration, suggesting sympathetic‒parasympathetic coactivation [16]. The second possible explanation is related to the perceived consequences of the stimulus; that is, disgust could be seen as a reaction to a threat of contamination or as a harm avoidance response; that is, a stress response. However, disgust is associated with nausea and revulsion feelings, contrary to fear’s fight-or-flight responses [11]. Thus, another possible explanation for the inconsistent findings is that HR decreases when a disgusting stimulus is presented (before there is a danger appraisal), but HR activation occurs when the stimulus is appraised as threatening or stressful [14]. In fact, disgust has been related to the activation of the anterior insula, whereas a threatening perception requires the activation of the amygdala and prefrontal lobe [28]. Therefore, neural activation can correlate (or not) with the physiological responses or the experiential state. Thus, a correlation has been described between the magnitude of subjectively experienced disgust and anterior insula activity, although these indexes did not correlate with sympathetic‒parasympathetic activity [28]. Alternatively, recently, some authors stated that, in nonclinical populations, perceived control is associated with reduced vasovagal symptoms when participants are presented with blood‒injury‒injection stimuli [29]. The interpretation of different cardiovascular responses being associated with different stimuli could reflect the level of survival influence that each stimulus supposes; that is, some stimuli can be related to injuries that can be interpreted as threatening, but others could be related to contamination feelings, producing a different cardiovascular response. Thus, there is a need for new studies that investigate the relationship between control and cardiovascular response in the light of the new knowledge related to vagal control.

Therefore, the general aim of the present study was to determine whether disgust is associated with decreases, increases, or no changes in the cardiovascular system. We have previously analyzed the role of vulnerability and emotional and cognitive variables in predicting the urge to wash evoked by a behavioral avoidance task (BAT) with a contamination-based stimulus, using three groups of participants (control group, subclinical contamination‒OCD, and subclinical checking‒OCD groups). Results showed that disgust was the primary emotional response to the task in all the groups, with the subclinical contamination‒OCD scoring higher. Moreover, the urge to wash was mainly predicted by the disgust experienced and the interpretation of disgust as threatening or dangerous [30]. In the present study, we will specifically analyze, with the same sample and protocol [30], the cardiovascular and emotional responses of subclinical contamination‒OCD and control groups during the BAT, in relation to their disgust feelings and emotional appraisals (valence, arousal and control/dominance). Specifically, we aimed to: (1) test whether participants with subclinical scores on contamination‒OCD have a different cardiovascular and psychological response than the control group during the confrontation with a potential contamination stimulus; (2) analyze the physiological response of disgust in relation to the psychological interpretation of the disgusting stimulus, depending on the sample.

## 2. Materials and Methods

### 2.1. Participants

The sample consisted of 403 voluntary participants (mean age 21.62 (SD = 3.91) years old; 85.80% women) who answered three screening measures: The revised Obsessive‒Compulsive Inventory (OCI–R) [31,32,33], which assesses distress associated with obsessive‒compulsive symptoms; the Beck Anxiety Inventory (BAI) [34,35], which measures anxiety symptoms; and the Beck Depression Inventory–II (BDI-II) [36,37], which measures depressive symptoms.

First, participants with severe depressive (BDI-II > 28) or anxious (BAI > 35) scores were excluded. Then, based on their scores on the screening measures, two groups were selected: (1) A high obsessive‒compulsive contamination symptom group (subclinical contamination‒OCD) composed of participants scoring above percentile 90 on the OCI-R washing scale (≥4) (*n* = 16; mean age 21.25 (SD = 1.61) years old; mean body index mass: 23.48 (SD = 3.81)); (2) a group of individuals who had low scores on the screening measures (control), below percentile 25 on the OCI-R subscales (≤2) and below 14 on mild depressive and anxious symptoms (BDI and BAI ≤ 13) (*n* = 14, mean age 23.13 (SD = 3.72) years old; mean body mass index: 22.11 (SD = 3.7)). In order to control for gender differences, both groups were composed of women because gender is a relevant factor in determining the OCD clinical presentation and course [38], and there is some evidence suggesting gender differences in some of the variables measured, such as disgust sensitivity [39,40], anxiety sensitivity [41], and contamination fears [39]. From the selected sample, three participants (two from the subclinical contamination‒OCD group and one from the control group) were eliminated for taking drugs that can influence cardiovascular functioning. Thus, the final sample for this study consisted of 27 women extracted from a large pool of undergraduate students: 14 in the subclinical contamination‒OCD group and 13 in the control group. All participants were drug-free (information provided by the participant). The use of analogue OCD samples has been considered relevant in understanding OC-related phenomena [42].

### 2.2. Procedure 

Participants who met the inclusion criteria for one of the groups were invited to participate in the second part of the research, which involved coming to the laboratory and participating in an experiment in exchange for a USB pen drive. Participants were met individually at the laboratory by one of the authors and signed an informed consent form. Then an HR monitor was placed on the individual, and she was invited to sit for 10 min to take baseline measurements. Afterwards, participants were involved in a seven-step behavioral avoidance task (BAT) involving a single stimulus (a garbage bag containing “dirty” underwear). Subjective and cardiovascular responses were registered throughout the task. After the BAT, participants were instructed to rest calmly in the chair in order to take HRV recovery measurements. Finally, participants were offered the chance to wash their hands, either with hand sanitizer or by going to the bathroom.

Experimenters were blind to the group assignment of each individual. The study was conducted in accordance with the Declaration of Helsinki, the protocol was approved by the Ethics Committee of the Universitat de València (Spain), and all individuals provided informed consent for inclusion before they volunteered to participate.

### 2.3. Behavioral Task

A behavioral approach/avoidance task (BAT) with seven steps was designed. Participants were asked to approach a garbage bag with underwear inside that was described as dirty. The stimulus was selected in order to conduct a BAT with a contamination-based stimulus evoking disgust, especially in subclinical contamination‒OCD participants [30]. As previously described [30], each step lasted 30 s, and the instructions were to: (1) approach the bag and look at it; (2) look at the bag and touch it with a paper tissue; (3) touch the bag with a finger (looking at the bag); (4) touch the bag with one hand (and look at it); (5) touch the bag with both hands (and look at it); (6) touch the bag (10 s) (and look at it) and touch your face with your hands (30 s); and (7) touch it (10 s), have a chocolate bar (or breadstick) in each hand (30 s), and eat the items. The BAT finished either when the participant refused to go on to the next step, or after the participant had eaten at least one chocolate bar/breadstick. Participants were instructed that they were free to refuse to do all or any part of the steps.

### 2.4. Measures 

Data for HR were continuously recorded during all the sessions using a Polar©RS800cx watch (Polar CIC, Bethpage, NY, USA), which consists of a chest belt for detection and transmission of the heartbeats and a receptor. The transmitter was situated on the chest belt, which was placed on the solar plexus. The Polar watch records RR intervals with a sampling frequency of 1000 Hz, providing a time resolution of 1 ms for each RR interval. The data collected by the Polar watch were downloaded and stored in the Polar ProTrainer 5TM program in the computer. HRV was analyzed with KUBIOS (Kuobio, Finland) software. Finally, time domain HRV measures were used to study the ANS activity. Specifically, HR (heart rate), RMSSD (root mean square of the successive differences), as time domain measures, and HFnu (normalized high frequency), as frequency domain measures were used to explore the role of vagal control [43].

During the BAT, subjective measures were assessed. After each step in the BAT, and prompted by the question, How did you feel while you were doing the task? Participants provided subjective ratings of subjective state emotions (anxiety and disgust), contamination feelings, urge to wash and urge to vomit responses (from 0 (none) to 10 (extreme)), and they appraised the valence, arousal, and dominance of the situation through a pencil-and-paper version of the self-assessment manikin [44] (SAM, a graphic nine-point scale of human-like figures). Using the scores on all these dimensions during the BAT steps, the following indices were calculated as average ratings: (1) anxiety, (2) disgust, (3) urge to vomit, (4) urge to wash, (5) contamination feeling, (6) negative emotional valence (e.g., unhappy, unsatisfied), (7) dominance/control (e.g., controlling, in control) and (8) activation (e.g., stimulated, excited) mean indexes.

### 2.5. Materials

A closed, black plastic garbage bag with clothes inside (subjects were told that it contained dirty underwear), disinfecting gel, chocolate bars, and breadsticks.

### 2.6. Data Reduction

First, RR registers were manually cleaned and filtered using the Kubios program and employing very low correction for 18 participants and strong correction for the rest. Once registers were filtered, and following the Task Force recommendations [43], the seven-step BAT was reduced to three periods of 5 min each. Thus, the first and second steps are considered the first measure of HRV (BAT1). The third, fourth, and fifth steps are considered the second measure of the BAT (BAT2). Finally, the sixth and seventh steps contain the last HRV measure for the analyses (BAT3). In addition to the HRV analyses, 5 min of baseline and recovery were obtained. Subjective measures during the BAT were reduced to three measurements in order to coincide with the three periods of HRV measurement.

### 2.7. Statistical Analyses

First of all, outliers were calculated using the three standard deviations method for variables measured one time and the Mahalanobis distance method *p* < 0.001 criterion for variables measured two or more times. No outliers were found in either group. The Kolmogorov‒Smirnoff test was used to check for normality. HR, RMSSD, anxiety, urge-to-vomit, urge-to-wash, contamination feeling, emotional valence, and dominance were normalized by the sqrt method.

One-way ANOVA was employed to explore sociodemographic or basal differences between groups. Only age was different between the groups (*F* (1, 26) = 4.808, *p* = 0.038), so it was used as covariate in all subsequent analyses. Repeated-measures ANCOVAs were performed with moment (five periods in the HRV variables: baseline, BAT1, BAT2, BAT3, and recovery; three periods in the subjective feelings: BAT1, BAT2, and BAT3) as the intrafactor variable, and group (contamination‒OCD and control) as the interfactor variable. Bonferroni tests were used for post hoc comparisons. To reduce the likelihood of type I error, we adjusted the degrees of freedom with the Greenhouse‒Geisser adjustment when appropriate to guard against violation of the sphericity assumption. Effect sizes (eta-squared) have been reported. Finally, Pearson correlations were performed to explore the relationships between HRV and subjective measures for each group and in the three BAT periods. All analyses were conducted with IBM SPSS Statistics (version 20) and Sigma Plot for figures.

## 3. Results

First, it is important to indicate that all the control group participants finished the seven steps of the BAT. However, only 10 of the subclinical contamination‒OCD participants finished the seven steps; the rest stopped after the sixth step. Thus, data regarding BAT3, which includes the sixth and seventh BAT steps for the subclinical contamination‒OCD group, include *n* = 10.

### 3.1. Cardiovascular Responses

**Figure 1:**
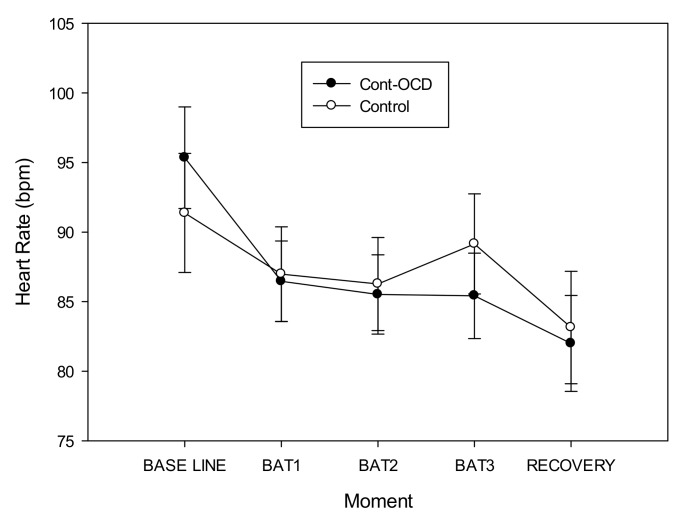
Heart rate mean levels and standard deviations during all the phases of the protocol (baseline, behavioral avoidance task (BAT1, BAT2, and BAT3), and recovery phases) in both groups (subclinical contamination‒OCD and control groups).

Our first objective was to test whether subclinical contamination‒OCD participants have a different response from the control group. Regarding cardiovascular response, ANCOVAs for HR showed a “moment*group” interaction (*F*(2.632, 42.108) = 3.150, *p* = 0.040, power = 0.652, *η*^2^_p_ = 0.164). Although post hoc analyses were not statistically significant, the data suggested that subclinical contamination‒OCD participants showed higher cardiovascular levels before the task began (baseline), but their cardiovascular levels were stable during the steps, whereas the control group showed increased HR levels at the end of the BAT (BAT3). After the recovery period, both groups finished with similar cardiovascular levels. Finally, although the ‘moment’ factor was not significant, post hoc analyses showed that HR decreased significantly (*p* < 0.001) from the first measure (baseline) to the final (recovery) measure (Figure 1).

In the case of parasympathetic measures, no between-group differences nor interaction were found. Moreover, no differences in HFnu were found depending on the group or interaction. 

### 3.2. Subjective Ratings

When cognitive ratings were analyzed to study differences between groups, ANCOVAs showed significantly higher scores in the subclinical contamination‒OCD group than control group on anxiety feelings (*F*(1, 24) = 14.692, *p* = 0.001, power = 0.957, *η*^2^_p_ = 0.380), urge to vomit (*F*(1, 24) = 5.593, *p* = 0.026, power = 0.622, *η*^2^_p_ = 0.189), urge to wash (*F*(1, 24) = 30.944, *p* = 0.000, power = 1.000, *η*^2^_p_ = 0.563), contamination feelings (*F*(1, 24) = 15.313, *p* = 0.001, power = 0.963, *η*^2^_p_ = 0.390), and disgust (*F*(1, 24) = 10.512, *p* = 0.003, power = 0.875, *η*^2^_p_ = 0.305), in the three moments of the BAT.

Furthermore, an effect of “moment” was found in both groups. The need to wash their hands increased significantly during the task (from BAT 1 to BAT 3) in both groups (*F*(1.35, 32.411) = 9.32, *p* = 0.002, power = 0.905, *η*^2^_p_ = 0.278). 

**Figure 2:**
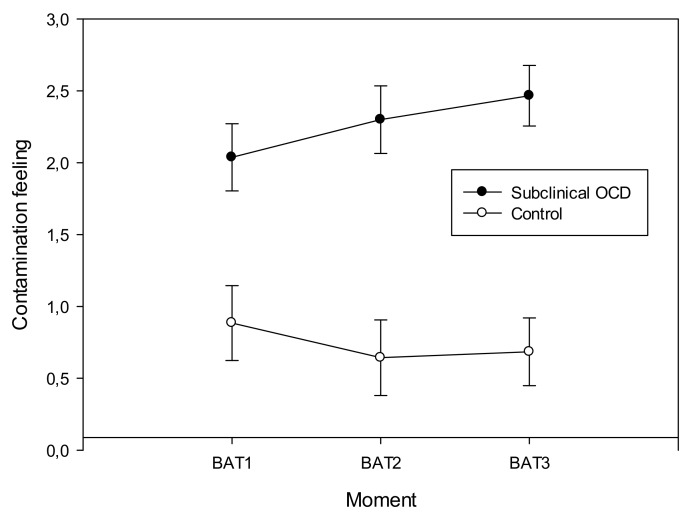
Contamination mean scores and standard deviation during the behavioral avoidance task (BAT) in both groups.

**Figure 3:**
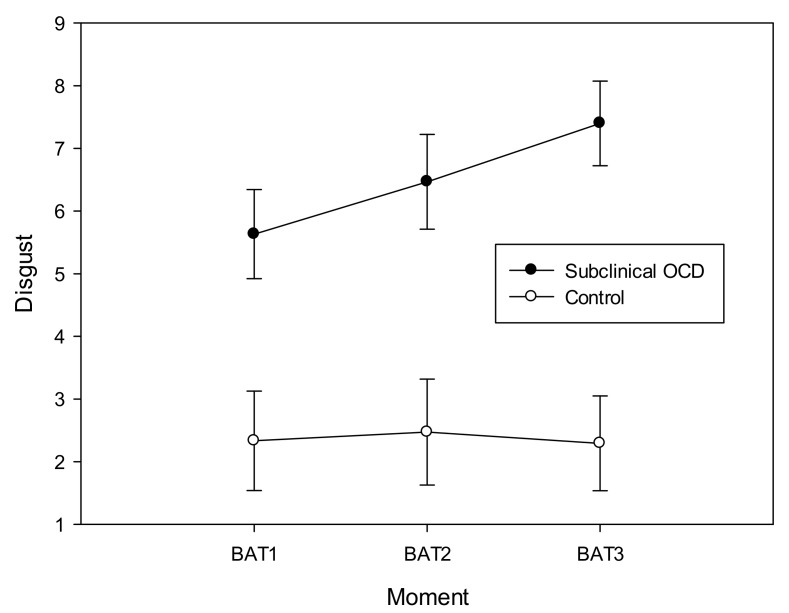
Disgust scores during the behavioral avoidance task (BAT) in both groups.

On the other hand, the “condition*moment” interaction was significant in the case of contamination (*F*(1.441, 34.579) = 4.961, *p* = 0.021, power = 0.682, *η*^2^_p_ = 0.171) and disgust (*F*(1.573, 37.743) = 6.756, *p* = 0.006, power = 0.840, *η*^2^_p_ = 0.220) feelings. Results showed that contamination and disgust feelings increased during the task, but only in the subclinical contamination‒OCD group (Figure 2 and Figure 3), having the group control stable scores in the three measures of the BAT.

When SAM emotional dimensions were analyzed, results revealed a “group” effect, showing the subclinical contamination‒OCD participants higher levels of negative emotional valence (*F*(1, 24) = 12.819, *p* = 0.002, power = 0.930, *η*^2^_p_ = 0.348) and dominance (*F*(1, 24) = 4.556, *p* = 0.043, power = 0.535, *η*^2^_p_ = 0.160) than control group. In addition, there was a decrease in activation during the task in both groups, although no significant differences were found (*p* > 0.05). Neither interactions, nor the effect of the “moment,” were found to be significant for SAM measures.

### 3.3. Relationships between Subjective Ratings and Cardiovascular Responses

Regarding the second objective, we analyzed the relationships between HRV and subjective measures. Only significant results will be described (all the correlations are depicted in Table 1 and Table 2).

In subclinical contamination‒OCD participants (Table 1), in BAT1, activation correlated significantly and positively with HFnu (*r*_14_ = 0.560, *p* < 0.05). In BAT1 and BAT2, dominance correlated significantly and positively with HFnu (*r*_14_ = 0.581, *p* < 0.05 and *r*_14_ = 0.533, *p* < 0.05, respectively). In addition, in BAT2 and BAT3, need to wash correlated positively with RMSSD (*r*_14_ = 0.545 and *r*_14_ = 0.581, respectively). In the control group (Table 2), at the end of the task (BAT3), Disgust correlated significantly (*p* < 0.05) with HRV indexes: positively with RMSSD and HFnu (*r*_10_ = 0.700 and *r*_10_ = 0.760, respectively) and negatively with HR (*r*_10_ = 0.760). Additionally, RMSSD correlated positive and significantly with disgust in BAT1 and BAT2 (*r*_10_ = 0.606 and *r*_10_ = 0.704, respectively) and with anxiety in BAT1 (*r*_10_ = 0.595).

## 4. Discussion

Our results show that the stimulus included in the BAT was a relevant contamination‒OCD stimulus provoking disgust, as previously reported [30]; in fact, the need to wash the hands increased in each of the steps of the BAT in both groups. In addition, it seems that the different steps in the task mainly evoked contamination feelings and disgust in the contamination‒OCD group, suggesting that these two variables represent the most relevant subjective experience. Moreover, the task evoked higher negative feelings in the OCD‒contamination group, suggesting that it is analogous to contamination‒OCD experiences. Additionally, four participants of the subclinical contamination‒OCD group did not finish the seven steps of the BAT. 

Furthermore, our results showed a reduction in HR in both the nonclinical group and the subclinical contamination‒OCD group, as previously found in high and low contamination fear groups [8], although at the end of the task HR increased slightly in the control group. However, HR was high at the beginning of the protocol. Moreover, none of the parasympathetic indexes presented differences between groups or in any of the BAT steps, consistent to what was found in other studies [14]. Therefore, our results do not show different responses between groups associated with disgust when young women are exposed to an object with no intrinsic contamination-related properties (in both subclinical contamination‒OCD and control samples). This result is not consistent with previous studies [7,8,22,45]). Taking into account that none of the vagal control indexes showed significant differences during the task, we interpret the cardiovascular response to disgust involve sympathetic and parasympathetic inputs, independently of the group; in this regard, we found lower levels of HR at the end of the disgust presentation (compared to the baseline), and this index is influenced by the combination of the sympathetic and parasympathetic input [14]. Although we cannot assert that HR decelerations were due to the disgusting stimulus, the literature has recently shown a HR deceleration associated with the RMSSD response in obsessive‒compulsive patients during exposure to a disgusting stimulus [7]. On the other hand, and considering that subclinical contamination‒OCD participants reported higher subjective negative responses to the stimulus than the control group (i.e., higher levels of disgust and contamination, increasing during the BAT, and higher levels of anxiety, urge to vomit, urge to wash, and contamination feelings from the first visual contact with the disgusting stimuli), our results seem to represent a dissociation between the reported subjective experience and the cardiovascular response, as recently found in nonclinical [8] and clinical OCD samples [7]. That is, whereas the subjective experience shows a clear feeling of disgust and anxiety, none of the cardiovascular indexes increased, but instead there was an HR deceleration (in both groups). In this regard, we speculate that these data could reflect a fractioning of the different response systems. Thus, whereas participants report subjective disgust feelings associated with the presentation and exposure to a potentially contaminated object, their cardiovascular systems did not react in this way. This result agrees with Rachman’s (1978) suggestion of emotions (fear) being “best construed as a set of loosely coupled components […] avoidance behavior, physiological reactivity and verbal/cognitive reports of subjective fear” (p. 239). In this sense, apart from differences between baseline and recovery, we expected differences in HRV during the development of the BAT, but none appeared. This result could imply that the BAT was subjectively efficient (increased the sense of disgust) but the cardiovascular system did not reflect the different increasing steps. The fact that not all the subclinical contamination‒OCD participants finished the task could account for the lack of differences. Related to this, the need to clarify the role of the sympathovagal balance in OCD was recently highlighted, and specifically the role of disgust, since treatments are focused on sympathetic activation and the presence of disgust could interfere with this activation [7]. In our case, disgust feelings seem to be predominant (compared to anxiety), as disgust and contamination feeling scores increased during the BAT, whereas anxiety scores were stable. We speculate that if the object of the BAT had not been disgusting (but stressful instead), it is possible that higher anxiety levels would have led to increases in HR; that is, a different stimulus (i.e., blood or a snake) would induce different psychological and physiological responses, and the relationships between them could be different. Another possible explanation could be that the different steps were not sufficiently different from the previous one, so participants did not reflect a significant difference between the increasing steps. Future studies should address this issue.

In light of these results, we looked for the reason for this dissociation. In our study, we measured, by means of the SAM, the degree of participants’ perceived control/dominance over their emotions during the BAT. Our results showed that contamination‒OCD participants scored higher on dominance appraisals than the control group. Although it seems surprising, due to the higher scores on the rest of the cognitive and emotional variables, we speculate that the perceived higher control could represent greater use of control strategies to cope with anxious situations and intrusions as it occurs in OCD populations [46]. Therefore, in spite of their nonpathological behavior, they would exert control actions (and regulate their emotions) in order to use adaptive behavior in their lives. Related to this, the need to study the effects of perceived control on clinical populations was recently emphasized [29]. In addition, it has been suggested that disgusting situations are associated with better proactive inhibition performance by using distracters as a strategy related to emotion regulation [47]. Emotion regulation models suggest that one of the primary strategies to regulate emotions is attentional deployment, which implies shifts in the attentional focus from emotional stimuli [45].

On the other hand, correlations give more information about the relationship between vagal control and the experience of disgust. Subclinical contamination‒OCD participants showed positive associations between activation and dominance and vagal control in the first two steps of the BAT. This result suggests that when the stimulus was presented, the subclinical contamination‒OCD group started up control mechanisms that could be translated into higher activation and dominance over the situation. These cognitive control mechanisms are related to vagal control. Following the neurovisceral hypothesis [17], we can consider that cardiovascular activity is controlled by the parasympathetic system through the activation of the prefrontal lobe, which has an important role in cognitive control [48]. Hence, the increase in activation (probably after watching the disgusting stimulus) turned on the reinforcement of the prefrontal lobe activity, increasing control over the heart (or inhibiting the cardiovascular response), and in turn decreasing HR. One possible explanation for this result is that cognitive control is exerted using distraction or other thought control mechanisms, as suggested previously [49], although this study has no measures to assert this speculation. However, these control strategies are frequently used by clinical and subclinical OCD participants to control their disturbing intrusions [46]. Moreover, as the BAT continues (in the two last steps), in the subclinical contamination‒OCD participants, the urge to wash their hands is related to the parasympathetic branch of the ANS, reinforcing the idea that the cognitive mechanisms to control the situation are associated with the parasympathetic response. On the other hand, in the control group, none of these relationships occurred. However, disgust correlated significantly with RMMSSD during all the BAT and in the last BAT step was positively associated with parasympathetic activity (RMSSD), vagal control (HFnu), and HR. This coincides with the fact that, in the last step of the BAT, the control group increased (not significantly) their cardiovascular activity compared to the subclinical contamination‒OCD group. Taking into account that disgust only correlates with ANS activity in the control group at the end of the BAT (where they have to touch their faces and eat food with “dirty” hands that have previously touched the bag), this finding could indicate that the control group was more vulnerable to the stimulus (feeling disgust) as they are not used to exerting control over disgusting stimuli. Disgust has been found to correlate with HR and RMSSD in OCD patients [7], similar to what occurred in our control group. Thus, we interpret that the absence of similar correlations in the case of the subclinical OCD group is due to the capacity for dissociation between what they are subjectively feeling and their physiological response. Another possible explanation for the correlations found in the last step of the BAT is that not all subclinical OCD participants reached the end of the BAT, reducing the number of samples and reducing the statistical power of these analyses.

As far as we know, this is the first study to analyze the cardiovascular response to an objectively nonthreatening situation that provokes disgust, especially in subclinical contamination‒OCD participants, trying to clarify the role of perceived control in disgust and its physiological correlates in contamination and washing OCD symptoms in young women. However, the results should be interpreted with caution. First, the number of participants was limited and we only considered young women, although they were selected from a larger sample using cutoff points widely used in the OCD literature. Nevertheless, in order to increase the reliability of the results, the experimental protocol was exhaustively controlled by taking different measures to explore the feelings experienced by the participants. Furthermore, in spite of participants having 10 min of adaptation before the protocol beginning, the higher levels of HR at the baseline make it difficult to interpret the cardiovascular measurements due to participants being stressed or anxious before the experiment started. Future studies should address these limitations in order to clarify the role of control in OCD symptoms, not only in young women but in other populations, especially in OCD patients.

## 5. Conclusions

Our results suggested that some cardiovascular responses in response to disgust could be associated with active cognitive control (dominance over the situation) in order to avoid the response to the disgusting stimulus interpreted as a potential contaminant (fear) in both subclinical contamination‒OCD and control young women. Moreover, studying the cardiovascular response to disgust probably does not reflect the pure reaction to the disgust emotion, but rather the interpretation of the disgust—that is, a more complex response that includes previous experience and cognitive (e.g., memory and attention bias) and emotional processes. In fact, previous results suggest that both the emotion and its appraisal as threatening influence the need to wash frequently, seen in OCD patients [30]. In this regard, our results suggest a dissociation between what participants feel and what the heart reflects, especially in subclinical contamination‒OCD participants. In conclusion, knowledge about the cardiovascular response to disgust could benefit from studies designed to improve the measurement of the cognitive processes involved in the disgust emotion. 

## Figures and Tables

**Table 1:** Pearson correlation coefficients in subclinical OCD-contamination group during the BAT.

		Anxiety	Disgust	Need to Vomit	Need to Wash	Contami-nation	Valence	Control	Activation
	HR	−0.079	0.131	0.271	−0.039	−0.330	−0.034	−0.423	−0.029
**BAT1**	RMSSD	0.144	−0.014	−0.356	0.138	0.132	0.018	0.324	0.066
	HFnu	−0.241	−0.410	−0.144	−0.348	−0.511	−0.103	0.581 *	0.560 *
	HR	−0.111	−0.043	0.318	−0.400	−0.486	0.136	−0.246	0.051
**BAT2**	RMSSD	0.150	0.102	−0.358	0.545 *	0.363	−0.013	0.205	−0.135
	HFnu	−0.284	−0.214	−0.113	0.092	0.047	−0.282	0.533 *	0.258
	HR	0.011	−0.212	0.409	−0.385	−0.466	0.202	−0.108	−0.017
**BAT3**	RMSSD	0.083	0.279	−0.307	0.581 *	0.264	−0.109	−0.002	−0.153
	HFnu	0.003	0.060	−0.085	0.105	0.183	−0.050	−0.019	−0.011

Note: * *p* < 0.05; BAT = Behavioral Avoidance Task; BAT 1 = BAT first and second step; BAT2 = third, fourth and fifth BAT steps; BAT3 = sixth and seventh BAT steps. HR = heart rate; RMSSD = Root mean square of the successive differences; HFnu = normalized high frequency. Data calculated with *n* = 14 for BAT1 and BAT2; and *n* = 10 for BAT3.

**Table 2:** Pearson correlation coefficients in control group during the BAT (Behavioral Avoidance Task).

		Anxiety	Disgust	Need to Vomit	Need to Wash	ContamiNation	Valence	Control	Activation
	HR	−0.395	−0.502	−0.319	−0.489	−0.407	0.232	0.060	0.326
**BAT1**	RMSSD	0.595 *	0.606 *	0.483	0.472	0.287	−0.266	−0.157	−0.504
	HFnu	0.445	0.539	0.530	0.258	0.340	−0.357	−0.043	−0.489
	HR	−0.128	−0.538	-	−0.623	−0.300	0.372	−0.172	0.151
**BAT2**	RMSSD	0.538	0.704 *	-	0.691	0.188	−0.409	0.092	−0.052
	HFnu	0.589	0.488	-	0.368	0.224	−0.103	−0.235	−0.315
	HR	−0.429	−0.661 *	−0.459	−0.383	−0.517	−0.276	0.445	0.774
**BAT3**	RMSSD	0.349	0.747 *	0.290	0.376	0.237	0.315	−0.353	−0.515
	HFnu	0.504	0.760 *	0.205	0.580	0.308	0.014	−0.140	−0.333

Note: * *p* < 0.05; - = missing data; BAT = Behavioral Avoidance Task; BAT 1 = BAT first and second step; BAT2 = third, fourth and fifth BAT steps; BAT3 = sixth and seventh BAT steps. HR = heart rate; RMSSD = Root mean square of the successive differences; HFnu = normalized high frequency. Data calculated with *n* = 13.
